# Aggressiveness Niche: Can It Be the Foster Ground for Cancer Metastasis Precursors?

**DOI:** 10.1155/2016/4829106

**Published:** 2016-07-14

**Authors:** Wael M. ElShamy, Abhilasha Sinha, Neveen Said

**Affiliations:** ^1^Cancer Institute, University of Mississippi Medical Center, Jackson, MS, USA; ^2^Department of Cancer Biology and Comprehensive Cancer Center, Wake Forest University School of Medicine, Winston-Salem, NC, USA

## Abstract

The relationship between tumor initiation and tumor progression can follow a linear projection in which all tumor cells are equally endowed with the ability to progress into metastasis. Alternatively, not all tumor cells are equal genetically and/or epigenetically, and only few cells are induced to become metastatic tumor cells. The location of these cells within the tumor can also impact the fate of these cells. The most inner core of a tumor where an elevated pressure of adverse conditions forms, such as necrosis-induced inflammation and hypoxia-induced immunosuppressive environment, seems to be the most fertile ground to generate such tumor cells with metastatic potential. Here we will call this necrotic/hypoxic core the “aggressiveness niche” and will present data to support its involvement in generating these metastatic precursors. Within this niche, interaction of hypoxia-surviving cells with the inflammatory microenvironment influenced by newly recruited mesenchymal stromal cells (MSCs), tumor-associated macrophages (TAMs), and other types of cells and the establishment of bidirectional interactions between them elevate the aggressiveness of these tumor cells. Additionally, immune evasion properties induced in these cells most likely contribute in the formation and maintenance of such aggressiveness niche.

## 1. Introduction

Tumor cells disseminate from primaries following a complex and stepwise process involving invading surrounding tissues, intravasation, and survival in the circulation, extravasation, and survival in a distant and foreign metastatic sites [1]. These monumental tasks require that tumor cells undergo several changes, such as transitioning from epithelial to mesenchymal (EMT), to be able to detach from primary site's extracellular matrix (ECM), migrate and invade surrounding tissues, and develop strategies to resist anoikis and the sheer forces within the circulatory system [2, 3]. Understanding these mechanisms and events that help generate such cells will benefit the design of therapies targeting disseminating cells and prevent cancer metastasis. Here, we propose an “aggressiveness niche” minimally defined as the necrotic/hypoxic core in tumors, within which recruited and activated mesenchymal stem cells (MSCs), tumor-associated macrophages (TAMs), and other stromal and inflammatory cells through bidirectional interactions entrain tumor cells to become metastasis precursors. These interactions also help generate conducive microenvironment for such entrainment.

## 2. The Role of Necrosis-Induced Inflammation in Aggressiveness Niche Formation

In aggressive tumors, the rate of proliferation exceeds that of neoangiogenesis leading to necrosis, especially within tumors cores. Unlike apoptotic cells, necrotic cells do not signal to nearby phagocytes to engulf and recycle them. Instead, intracellular content, including damage-associated molecular pattern (DAMP) materials, spills into the microenvironment leading to increase in inflammation within these cores.

**Figure 1:**
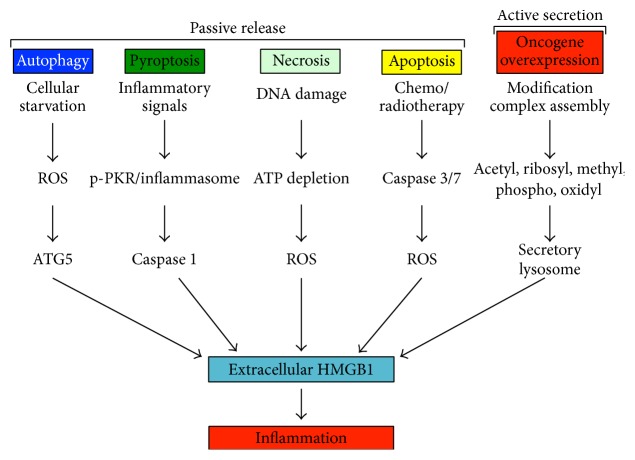
HMGB1 and inflammation. The forces contributing to the passive release and the active secretion of HMGB1 from tumor cells within the aggressiveness niche.

High mobility group binding (HMGB1) protein is well-studied DAMP normally bound to chromatin [4]. HMGB1 can be passively released from necrotic, autophagic, and apoptotic cells [5, 6] or actively secreted from oncogene-activated tumor cells [7] (Figure 1). Modifications such as methylation, glycosylation, ribosylation, and acetylation promote release from chromatin and the cytokine function of HMGB1 [8–11].

Extracellular HMGB1 binds in either autocrine or paracrine manner to several cell surface receptors, including receptor for advanced glycation end products (RAGE) and toll-like receptors (TLRs) [12–15]. Binding to these receptors activates proinflammatory signaling pathways, such as the NF-*κ*B, IFN regulatory factor-3 (IRF3), phosphoinositide 3-kinase (PI3K), and inflammasome to induce proinflammatory cytokine release into the microenvironment [16, 17]. Therefore, extracellular HMGB1 sustains an inflammatory microenvironment within tumors that supports tumor growth, invasion, and metastasis [18–22]. Indeed, several studies showed that inhibiting HMGB1-RAGE or HMGB1-TLR4 interactions suppresses inflammation, tumor growth, and metastasis in animal models [18, 23]. Additionally, in the clinic, expression of RAGE or TLR4 is closely associated with invasion and metastasis [21, 22]. Accordingly, neutralizing HMGB1 antibody or RAGE knockdown inhibited tumor angiogenesis and metastasis* in vitro* and* in vivo* [22]. Furthermore, chemotherapies promote cell death in tumors concurrently with sequestration of HMGB1 in the nucleus, preventing its release even if necrotic death ensues.

Another powerful factor spilled out of necrotic cells is ATP [24]. ATP activation of the P2X7 purinergic receptor on tumor cells in autocrine or paracrine fashion leads to fall in the intracellular potassium level, which triggers the oligomerization of the “inflammasome” [25]. The inflammasome contains proteins, such as cryopyrin or nucleotide-binding domain and leucine-rich repeat containing protein 3 (NLRP3) and procaspase 1. The inflammasome processes procaspase 1 into an active cysteine protease “caspase 1” [26]. Caspase 1 then binds and cleaves IL-1*β* precursor converting it to the active secreted form [27]. Caspase 1 is constitutively active in highly metastatic human cancers, especially those with mutation in cryopyrin [28, 29]. The activation of inflammasomes and their downstream targets contribute to innate and adaptive immunologic defense mechanisms by the regulation of several different and partially opposing pathways [30].

The adaptive immune system is divided into CD4^+^ and CD8^+^ T-cell lineages. Activation through unique T-cell receptors (TCRs) and costimulation by antigen-presenting cells (APCs), such as dendritic cells (DCs), rapidly enhance T-cells proliferation and differentiation into effector cells. Effector CD4^+^ T-cells develop as interferon-*γ* (IFN-*γ*) producing T helper cells (Th_1_), IL-4/IL-13 producing Th_2_ cells, IL-10 producing regulatory T (T_reg_) cells, and IL-17 producing Th_17_ cells. CD8^+^ T-cells are mainly considered cytotoxic T lymphocytes (CTLs) and produce cytotoxic granules that kill cancerous cells. Extracellular HMGB1 induces apoptosis in DCs, thus suppressing CD8^+^ T-cells function and enhancing T_reg_ function and diminishing host anticancer immunity within the necrotic core [31, 32], which lead to tumor progression [33]. Extracellular IL-1*β* induces accumulation of myeloid-derived suppressor cells (MDSCs) that impairs NK cells development and functions* in vitro* and* in vivo* [34]. MDSCs contribute to tumor progression and growth by suppressing antitumor immune responses via blocking CD4^+^ and CD8^+^ T-cells activation [35]. Taken together, these findings highlight the potential important role of necrosis in the development of the aggressiveness niche, in which an inflammatory environment provides an immune evasion response leading to cancer progression. In fact, recent clinical trial showed great efficacy for the anti-IL-1*β* monoclonal antibody “anakinra” [36].

## 3. The Role of Hypoxia-Induced Adaptation in Aggressiveness Niche Formation

In a fast-growing tumor, the diffusion distance from the existing vascular supply increases resulting in hypoxia [37, 38]. Hypoxia affects tumors in many ways including enhancing cell growth rate, neovascularization, metastasis, and resistance to treatment.

In cancer tissues, large areas of hypoxic tissue and concentration of the hypoxic markers, such as CAIX and HIF-1*α* exist around necrotic regions. HIF-1 family of basic helix-loop-helix transcription factors includes HIF-1*α*, HIF-2*α*, and HIF-3*α* [39]. Only HIF-1*α* is destabilized and degraded under normoxic conditions, whereas, under hypoxic conditions, it is stabilized and translocated to the nucleus to heterodimerize with the constitutively expressed HIF-1*β* [40, 41]. The HIF-1*α*-HIF-1*β* complex through binding to HIF response elements (HREs) in the promoter regions of important adaptive genes activates their transcription [42–46].

Hypoxia also promotes metabolic shift and lowers the pH within the aggressiveness niche impeding the adaptive immune response and acts to recruit immune suppressive cells, such as MDSCs, T_regs_ that significantly reduce CD4^+^ T-cell proliferation, CD8^+^ T-cell, and natural killer (NK) cells cytotoxicity [47–50]. The anaerobic conditions within the aggressiveness niche ferment the pyruvate produced by glucose metabolism in tumor cells into acidic lactate, which helps altering the metabolism in the niche in what is called “Warburg effect” [51]. The Warburg effect also suppresses the maturation of antigen-presenting cells (APCs) that activate naïve T-cells [52]. Taken together, these findings highlight the potential important role of hypoxia in generating an immune suppressive microenvironment and together with the necrotic microenvironment maintain an immune evasion response that promotes cancer progression.

## 4. The Role of MSCs in Aggressiveness Niche Formation

MSCs are primitive cells mobilized from the bone marrow to sites of hematopoiesis, inflammation, injury, and solid tumors [53–56]. Within these sites MSCs differentiate to give rise to cells of many lineages, including muscle, bone, fat, and cartilage lineages [57, 58]. Recent data also point to the fact that, within tumors, MSCs can differentiate into carcinoma-associated fibroblasts (CAFs) [59–66]. CAFs' role in enhancing tumor growth, progression, metastasis, and therapeutic resistance has been shown in many cancers [67–69].

The aggressiveness niche resembles, to a great extent, tissues undergoing chronic inflammation [70]. This causes immune response leading to homing of MSCs to aggressiveness niche in response to chemotactic factors, such as the monocyte chemotactic protein-1 (MCP-1) [71], cyclophilin B, the hepatoma-derived growth factor (HDGF) [72], and IL-6 [73], and activation of intracellular signaling in MSCs, such as STAT3. Cancer cells, especially those with cancer stem-like cells (CSCs) activity [74, 75], such as triple negative breast cancer cells (TNBCs), secret effector cytokines, including IFN-*γ*, TNF-*α*, and IL-1*β* that activate MSCs immunosuppressive role [76, 77]. Activated MSCs then produce many immune-modulatory molecules such as hepatocyte growth factor (HGF), transforming growth factor-*β* (TGF-*β*), prostaglandin E2 (PGE2), IL-10, and inducible nitric oxide synthase (iNOS) [78–82]. These cytokines suppress IFN-*γ* production from Th_1_, promote IL-4 secretion from Th_2_, and increase T-cells polarization more towards TGF-*β*-expressing T_reg_ cells, rather than IL-17-expressing Th_17_ cells [78–82], thus generating immunosuppressive environment that promotes tumor cells aggressiveness within the niche.

Furthermore, MSCs express the major histocompatibility complex (MHC) class I but lack class II MHC along with the costimulatory molecules CD80, CD86, and CD40 [83, 84]. MSCs can suppress T-cell proliferation and activation in response to allogeneic antigens [82], inhibit B-cell proliferation, differentiation, and antibody generation [85], interfere with DCs maturation and function [86, 87], recruit CD8^+^Foxp3^+^CD25^+^ T_reg_, and promote their proliferation. Taken together, these studies demonstrate the profound effect of MSCs in exacerbating tumor progression through bidirectional interactions with tumor cells [67] or indirectly through effects on the tumor microenvironment [63, 66].

## 5. The Role of TAMs in Aggressiveness Niche Formation

Monocytes also originate from the bone marrow, where they enter the peripheral blood and infiltrate into tumors [88]. In breast cancers, nearly half the tumor mass consists of tumor-associated macrophages (TAMs). In tumors, TAMs accumulation associates with disease progression and is often correlated with poor prognosis [89]. Within tumors, monocytes can differentiate into specialized phagocytes M1-macrophages that engulf and digest dead and tumor cells or into pro-tumor M2-polarized macrophages. M1-macrophages primed by IFN-*γ* could be activated by tumor necrosis factor-*α* (TNF-*α*) or by activation of toll-like receptors (TLRs) via exposure to microbes or microbial products such as bacterial LPS [90]. M1-polarized macrophages could also function as antigen-presenting cells [91] and secrete high levels of inhibitory interleukin-12 (IL-12) and IL-23 cytokines [92]. On the other hand, in the presence of cytokines, such as IL-4 and IL-13, macrophages differentiate into immunosuppressive M2-macrophages characterized by IL-10 production that promotes tumor progression [90, 93].

Like MSCs, TAMs generally accumulate in hypoxic areas of the tumor. MSCs skew macrophages towards the M2-polarization* in vivo* leading to increase in IL-10 production and decreased proinflammatory cytokine and NO production in tumors, which leads to immunosuppressive environment instead of initiating T-cell-mediated immune responses within tumors [79, 86]. Prostaglandin E2 (PGE2) constitutively produced by human MSCs suppresses IL-6 and TNF-*α* expression in macrophages. In addition, neutralizing antibodies to IL-6 and granulocyte macrophage-colony stimulating factor (GM-CSF) showed that these cytokines synergistically promote human gingiva-derived MSC-mediated promotion of the M2 phenotype in macrophages [89–93].

Other immune system entities such as mast cells, neutrophils, NK cells, and dendritic cells (DCs) contribute to breast tumor progression, especially through the release of proinflammatory cytokines. MSCs also reduced inflammation promoted by mast cells and were resistant to cytotoxicity by NKs. Neutralization of PGE2 and transforming growth factor-*β* (TGF-*β*), both thought to contribute to MSC immunosuppression, overrode MSC-mediated suppression of NK proliferation. MSCs also interfere with DC maturation, IL-12 production, and migration to lymph nodes* in vivo*, leading to insufficient T-cell priming in the lymph nodes [31].

## 6. Concluding Remarks

There is a complicated interplay between cancer and the host immune system. Understanding this interplay and the mechanisms by which tumors evade immune control should identify new and innovative therapeutic strategies. Reversing these immune evasion strategies could strengthen the adoptive immune system as a promising tool for cancer therapy. Additionally, understanding all the mechanisms tumors use to establish growth and subsequently metastasize, in part through evasion of immune surveillance, for example, the role of MSCs in such evasion, could help in generating combinatorial therapies that provide therapeutic efficacy by preventing the suppressive effect of MSCs and activating the antitumor effect of the immune system. It should be noted, however, that experimental evidences that support the notion that cells within the niche are those that form the metastasis are still lacking.
